# Times Are Harsh, Be Kind to Yourself! Anxiety, Life Satisfaction, and the Mediating Role of Self-Compassion

**DOI:** 10.3389/fpsyg.2022.915524

**Published:** 2022-06-07

**Authors:** Alexandra Maftei, Georgiana Lãzãrescu

**Affiliations:** Department of Educational Sciences, Faculty of Psychology and Educational Sciences, Alexandru Ioan Cuza University, Iaşi, Romania

**Keywords:** prospective anxiety, inhibitory anxiety, life satisfaction, intolerance of uncertainty, self-compassion

## Abstract

The present research aimed to explore the indirect effect of self-compassion on the relationship between two dimensions of intolerance of uncertainty (i.e., prospective and inhibitory anxiety) and life satisfaction. One hundred sixty-four Romanian adult participants formed our sample from the country’s eastern side. Their ages ranged between 18 and 61 (*M* = 23.45, *SD* = 7.70, 72% females). The study was conducted in 2021, when the Delta wave of COVID-19 was at its peak. Our findings suggested significant negative associations between prospective anxiety, self-compassion, and life satisfaction. A similar negative significant association was suggested between inhibitory anxiety and self-compassion. Age did not significantly correlate with any of our primary variables. Mediation analyses indicated a total mediating effect of self-compassion on the relationship between participants’ prospective and inhibitory anxiety and their life satisfaction. We discuss the implications of our findings, considering their relevance for therapeutical interventions aimed to promote psychological wellbeing when facing adversity.

## Introduction

During the past two challenging years, the world has been through many difficult moments, many of them caused by the COVID-19 breakout. As a result, high levels of uncertainty marked these past COVID-19 years, along with fear, worry, anxiety, and general psychological distress (e.g., Deniz, 2021; Millroth and Frey, 2021; Voitsidis et al., 2021; Del-Valle et al., 2022). Since its outbreak, the COVID-19 pandemic has infected more than 490,000,000 people worldwide, and over 6.1 million COVID-19 deaths have been reported (Worldmeter, 2022). There have been over 2.8 million infections and more than 65,000 coronavirus-related deaths in Romania since the pandemic began. More importantly, Romania had the highest COVID mortality rate in the world during the Delta wave, i.e., in the fall of 2021 (Gherasim, 2021). For example, at the beginning of November 2021, the number of COVID-related deaths in Romania was around 600 per day, killing one Romanian every 5 min. Romania was also the first European country to lift the pandemic restrictions in 2021 and relax other measures, but next-to-last regarding vaccination rates (Gherasim, 2021).

A growing number of studies have already documented the various ways that such challenging times might activate both constructive and destructive coping mechanisms, as well as the associated protective (e.g., self-compassion) and risk factors (e.g., intolerance of uncertainty) when dealing with adversity (e.g., Kunzler et al., 2021; Racine et al., 2022). Thus, in these turbulent times, marked by unpredictability and stress (Jayaram et al., 2022), we consider it important to explore how intolerance of uncertainty might contribute to the variations in people’s life satisfaction. Furthermore, we aimed to explore how self-compassion might mediate the relationship between the two dimensions of intolerance of uncertainty (i.e., prospective and inhibitory anxiety) and life satisfaction.

### Intolerance of Uncertainty

Intolerance of uncertainty (IU) refers to an adverse cognitive, emotional, or behavioral reaction to an uncertain situation or one’s “dispositional incapacity to endure the aversive response triggered by the perceived absence of salient, key, or sufficient information, and sustained by the associated perception of uncertainty” (Carleton, 2012, p. 31). Research generally underlines that individuals with high levels of IU perceive the uncertain situation as threatening, undesirable, and upsetting, even if the probability of its appearance is low (e.g., Bottesi et al., 2016). Furthermore, the various adverse effects of one’s high IU are linked, among others, to emotional difficulties, anxiety, and depression (e.g., Deniz, 2021). Furthermore, IU might also limit the ability to adapt and cope (McEvoy and Mahoney, 2012), contributes to lower life satisfaction (Karataş and Tagay, 2021), and, generally, to various psychopathologies and maladjustment (e.g., Luhmann et al., 2011; Boswell et al., 2013; Chen et al., 2018; Osmanaðaoðlu et al., 2018).

Several factors seem to contribute to the development and maintenance of IU. For instance, the Intolerance of Uncertainty Model of GAD (IUM) proposed by Dugas et al. (1998) suggested that four primary factors seem to be relevant in this specific context, i.e., IU, positive beliefs about worry, negative problem orientation, and cognitive avoidance. Similarly, Ouellet et al. (2019) highlighted that positive beliefs about worry, cognitive avoidance, and negative problem orientation significantly contribute to IU. For example, individuals might adopt a positive perspective about their concerns, believing that worries are helpful and preparing them to avoid anything wrong (i.e., positive beliefs about worry). Thus, positive beliefs about worries might work as a coping or protective mechanism against potential adversities (Ouellet et al., 2019). In addition, cognitive avoidance, i.e., the effort to eliminate intrusive images and thoughts related to possible adverse events, might enhance anxiety by preventing exposure to the possible threat. Finally, negative problem orientation, i.e., the general tendency to be pessimistic about one’s problem-solving resources, determines the perceptiveness of relatively minor problems as a serious threat. Thus, the remaining “unresolved” issues further enhance anxiety (Ouellet et al., 2019).

IU was explored both as a unitary construct (McEvoy et al., 2010) and as a binary construct liked to anxiety, i.e., prospective and inhibitory anxiety (Birrell et al., 2011; Thibodeau et al., 2013). In the present study, we explored IU using the binary approach. The first dimension (i.e., prospective anxiety) represents the tendency to focus on future events to prepare for potential threats. Previous studies found that prospective anxiety, as a dimension of IU, might be related to some disorders such as generalized anxiety disorder and obsessive-compulsive disorder (Thibodeau et al., 2013). On the other hand, inhibitory anxiety describes the tendency to inhibit one’s response in an uncertain situation. Individuals high on inhibitory anxiety experience might feel incapable of productively responding when dealing with an uncertain situation (Fourtounas and Thomas, 2016). Similar to prospective anxiety, the inhibitory dimension of IU was linked to several symptoms of social anxiety disorder, panic disorder, and depression (McEvoy and Mahoney, 2011; Thibodeau et al., 2013). We chose this particular binary approach given the ambiguity of the COVID-19 context, which requires a more comprehensive view of the two dimensions of IU, as previous studies suggested (e.g., Wu et al., 2021).

### Self-Compassion

The growing interest in the relationship between compassion and mental health outcomes is generally due to the significant evidence highlighting the essential role of compassion (and self-compassion) in people’s overall wellbeing, life satisfaction, and affective experiences (MacBeth and Gumley, 2012). Goetz et al. (2010) defined compassion as “a distinct affective experience whose primary function is to facilitate cooperation and protection of the weak and those who suffer” (p. 351). Self-compassion also involves “being touched by and open to one’s own suffering, not avoiding or disconnecting from it, generating the desire to alleviate one’s suffering and to heal oneself with kindness” (Neff and Germer, 2017). Contrary to self-esteem, self-compassion is based on kindness, understanding, support, and forgiving feelings toward ourselves, including adverse situations or personal failures (Souza and Hutz, 2016).

Self-compassion also comprises three related constructs, i.e., self-kindness, common humanity, and mindfulness (Gilbert, 2009; Neff, 2011; Neff and Knox, 2016). Each of these related constructs has two different dimensions, and the second one is the opposite of the first: self-kindness vs. self-judgment, common humanity vs. isolation, and mindfulness vs. overidentification or avoidance (Barnard and Curry, 2011; Neff, 2011). Self-kindness involves emotionally or mental acts of kindness, such as being caring and understanding, supportive, patient, sensitive, warm, and forgiving to all aspects of ourselves (e.g., actions, feelings, thoughts, or impulses) (Allen and Leary, 2010; Barnard and Curry, 2011; Neff, 2011). Being kind and being aware and accepting that we are imperfect represent an essential characteristic of self-kindness (Neff, 2011).

Another facet of self-compassion, common humanity, refers to the importance of recognizing that we are prone to make mistakes, fail, loss, or rejection, and these are natural parts of our lives (Allen and Leary, 2010). However, when experiencing pain, frustration, or hard times, people may feel that their experience is unique, feeling disconnected from the others around them. In other words, when people feel isolated, acknowledging that they are not the only ones suffering or having a hard time is essential to reduce their feelings of isolation and loneliness (Allen and Leary, 2010; Neff, 2011). Finally, when we consider self-compassion through mindfulness vs. overidentification or avoidance, we refer to awareness, attention, and acceptance of the present moment and experience (Barnard and Curry, 2011). Through this facet of self-compassion, people can experience a balanced way of accepting their lives and ignoring the less positive aspects. Neff (2011) suggested that people who are low in self-compassion might focus on the negative parts of some situations and get stuck there, but those who act in a self-compassionate (mindfulness-based) way might manage it more positively and effectively (Allen and Leary, 2010).

Since the COVID-19 outbreak, self-compassion has been explored in a growing number of studies. Most of them suggested that self-compassion buffers the adverse mental health effects of the pandemic (Lau et al., 2020). Furthermore, Deniz (2021) suggested that individuals high in self-compassion reported lower IU, which further decreased the COVID-19 related psychological distress.

### Life Satisfaction and Self-Compassion

Though it variates from one life stage to another and from one culture to another (Loewe et al., 2014), life satisfaction generally refers to the positive evaluation of various aspects of one’s life, such as family, career, friends, or health. Although correlated with subjective wellbeing, life satisfaction is a different construct. Life satisfaction comprises the cognitive, global evaluation of one’s quality of life (as a whole) (Pavot and Diener, 2008); meanwhile, subjective wellbeing is a multifaceted construct, with both affective, and cognitive components (Diener et al., 1999).

Since the COVID-19 pandemic, many studies have explored people’s psychological states and outcomes, and some have investigated the long-term effects of the COVID-19 crisis on people’s life satisfaction. For example, the longitudinal investigation conducted by Benke et al. (2022) suggested that participants’ life satisfaction decreased from baseline (May 2020) to 6 months (November 2020) and 1-year follow-up (June 2021). Similar effects were reported by Kozina et al. (2022) in their related 3-wave longitudinal research. These parallel findings suggested a similar pattern of decrease in life satisfaction since the beginning of this worldwide health crisis.

Self-compassion promotes kindness, understanding, and acceptance and develops adaptive coping strategies. At the same time, life satisfaction refers to a generally positive evaluation of an individual’s life (Yang et al., 2016). Thus, researchers who explored the relationship between these two constructs generally expected that individuals high in self-compassion might also have a higher level of life satisfaction. Self-compassion seems to increase an individual’s life satisfaction by being kind, supportive, patient, forgiving, and understanding with themselves. Other studies suggested that lower self-compassion, self-efficacy, and mindfulness significantly predict anxiety (Soysa and Wilcomb, 2015). Finally, Kim and Ko (2018) conducted a study about the impact of self-compassion on mental health, quality of life, and life satisfaction, and their results suggest that participants who scored lower on self-compassion and higher on self-judgmental reported being more isolated. Moreover, results suggested that a low level of self-compassion was negatively related to higher depression.

### The Present Study

Since self-compassion seems to reinforce one’s positive re-evaluation and promote active coping behaviors, self-compassion also contributes to lower levels of IU. For example, Tang (2019) suggested that self-compassion was a significant negative predictor of IU, as well as mindfulness therapy (Asli Azad et al., 2019), which is a well-known facet of self-compassion (Barnard and Curry, 2011). Also, since IU challenges one’s positive psychological state and general wellbeing, triggering stress (Satici et al., 2020), it can also decrease life satisfaction, especially during challenging times, such as the pandemic, when uncertainty increases fear of COVID-19 (Satici et al., 2020; Rettie and Daniels, 2021; Maftei and Holman, 2022).

Deniz (2021) suggested that self-compassion might predict happiness through intolerance of uncertainty and fear of COVID-19 (in a sequential manner). However, in the present study, we explored a mediation model using self-compassion as the mediating variable between the two facets of IU, i.e., prospective and inhibitory anxiety and life satisfaction. Based on the previous findings exploring the relationship between the overall measure of IU, as well the more recent findings (i.e., Deniz, 2021) concerning the dynamics of these relationships during the pandemic, we assumed that we would find a significant indirect effect of self-compassion on both facets of IU and life-satisfaction.

Previous studies (e.g., Deniz, 2021) made it clear that IU might decrease people’s life satisfaction, but the dynamic concerning both facets of IU, through self-compassion, is not yet clear at the peak of the deadliest pandemic wave in Romania. Based on the previous findings exploring the relationship between IU, life satisfaction, self-compassion, and the dynamic multi-directional ways of these relationships during the COVID-19 pandemic (e.g., Barnard and Curry, 2011; Asli Azad et al., 2019; Tang, 2019; Satici et al., 2020; Deniz, 2021; Rettie and Daniels, 2021), we explored the potential significant indirect effect of self-compassion on both facets of IU and their link with life satisfaction.

Furthermore, as we chose a more generalized perspective related to the measurement of our primary variables, we did not include a specific variable related to COVID-19, as in Deniz’s (2021) study.

## Methods

### Participants and Procedure

Our sample included 164 adult participants from the eastern side of Romania. Their ages ranged between 18 and 61 (*M* = 23.45, *SD* = 7.70, 72% females). The research protocol was designed following the ethical requirements specific to the faculty where the authors are affiliated. The participants added their answers using a web-based platform in the fall of 2021, during the peak of the COVID-19 Delta wave in Romania. We used a snowball sampling technique. The survey was advertised using students’ social media groups, from all educational levels (Bachelor’s, master’s, doctoral and post-doctoral levels), from the university where the authors are affiliated, and who invited further potential participants. The only inclusion criterion was related to age (> 18).

All participants voluntarily participated in the study and gave written informed consent, following the 2013 Declaration of Helsinki. The time needed to answer all the questions was around 20 min. All participants were informed that their information was anonymous and that their answers would remain confidential. Also, they were told that there were no right or wrong answers, that sincerity was the most significant characteristic of their participation, and that they could leave the study at any time.

### Measures

#### Intolerance of Uncertainty

We used the 12-item Intolerance of Uncertainty Scale developed by Carleton et al. (2007). The scale’s items are scored on a 5-point Likert scale ranging from 1 (not at all characteristic of me) to 5 (entirely characteristic of me). Items measure two dimensions of IU: prospective anxiety (e.g., “It frustrates me not having all the information I need”), and inhibitory anxiety (e.g., “The smallest doubt can stop me from acting”). In the present study, we computed a total score for each factor. Cronbach’s Alpha indicated satisfying reliability values for both dimensions (i.e., 0.83 for prospective anxiety and 0.80 for inhibitory anxiety). Higher scores indicated higher levels of anxiety.

#### Self-Compassion

We further used the Self-Compassion Scale-Short Form (Raes et al., 2011), a 12-item self-report measure using a 5-point Likert scale (ranging from 1—almost never, to 5—almost always). The instrument comprises six separate dimensions of self-compassion, as well as an overall measure, which we used in our research. Example items include “I try to be understanding and patient toward those aspects of my personality I don’t like” and “I try to see my failings as part of the human condition”). Cronbach’s Alpha indicated satisfying internal consistency (0.69). Higher scores indicated higher levels of self-compassion.

#### Satisfaction With Life

Participants’ satisfaction with life was measured using the Satisfaction with Life Scale (Diener et al., 1985). The scale comprises five items that measure respondents’ level of satisfaction on a 7-point Likert scale, ranging from 1 (strongly disagree) to 7 (strongly agree). Example items include “In most ways my life is close to my ideal.” Cronbach’s Alpha indicated good internal consistency −0.86. Higher scores indicated higher levels of satisfaction.

#### Demographics

Finally, a demographic scale assessed participants’ age and gender.

We employed the back-translation procedure to check the consistency of the quality of the translated research instruments (which were presented in Romanian), and we found no discrepancies (Tyupa, 2011).

### Overview of the Statistical Analyses

We used the 26 version of the IBM SPSS statistical package and Hayes (2013) PROCESS macro to analyze our data. Our data had no missing values because all the items were set as required (Kyriazos, 2018). However, all participants who accepted to voluntarily participate filled all the answers, so there were no partially filled scales. We first conducted a preliminary analysis to assess the descriptive statistics of the primary variables. We further computed zero-order bivariate correlations between the study’s main variables. Next, we investigated the potential mediating effect of self-compassion on the relationship between prospective and inhibitory anxiety and life satisfaction using model 4 (95% confidence interval (CI); 5,000 resampled samples).

## Results

The descriptive statistics (i.e., means, standard deviations, minimum, and maximum scores) for the primary variables, *t*-test results (gender), and zero-order correlations are presented in Table 1. Normality distributed variables should have Skewness between -1 and 1 and Kurtosis between -3 and 3 (Kim, 2013), and the values in our sample indicated normal distributions. *T*-test results did not suggest any gender-related differences among the primary variables (all *p*s > 0.05).

**TABLE 1:** Descriptive statistics and *t*-test results (gender) for the primary variables (*N* = 164).

	M	SD	Min	Max	Skewness	Kurtosis	t-test
1. Prospective anxiety	18.46	5.77	7	35	0.20	–0.08	–1.25
2. Inhibitory anxiety	13.14	4.30	5	25	0.09	–0.29	–1.24
3. Self-compassion	2.90	0.48	0.92	4.25	–0.30	2.03	0.76
4. Life satisfaction	23.19	5.94	5	35	–0.49	0.35	–0.37
5. Age	23.45	7.70	18	61	−	−	−

The Pearson correlation analysis indicated significant negative associations between prospective anxiety and self-compassion (*r* = 0.39, *p* < 0.001) and life satisfaction (*r* = −0.20, *p* = 0.01). Thus, the higher the prospective anxiety, the lower participants’ self-compassion and satisfaction with life. A similar negative significant association was suggested between inhibitory anxiety and self-compassion (*r* = −0.36, *p* < 0.001). Age did not significantly correlate with any of the variables (see Table 2).

**TABLE 2:** Zero-order correlations among the primary variables (*N* = 164).

	1	2	3	4
1. Prospective anxiety	−			
2. Inhibitory anxiety	0.70**	−		
3. Self-compassion	−0.39**	−0.36**	−	
4. Life satisfaction	−0.20**	–0.11	0.32**	-
5. Age	–0.20	–0.03	0.02	0.05

**p < 0.001.

Based on the results from the correlation analyses, we further Model 4 (95% CI, 5,000 bootstrapped samples) to investigate the potential mediating role of self-compassion on the relationship between participants’ prospective and inhibitory anxiety and life satisfaction.

**FIGURE 1:**
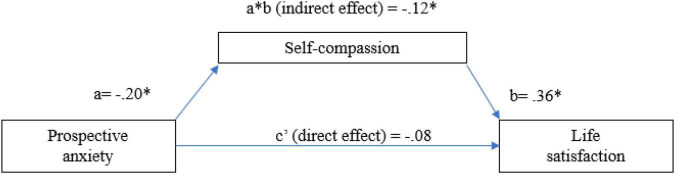
The mediating effect of self-compassion on the relationship between prospective anxiety and life satisfaction. The values represent unstandardized coefficients. *p < 0.05; *N* = 164.

*a. The mediating role of self-compassion on the relationship between participants’ prospective anxiety and life satisfaction.* The total effect of participants’ prospective anxiety (i.e., without considering the mediator) was significant; *b* = −0.20, SE = 0.07, *t(*161) = −2.60, *p* = 0.009, 95%CI [−0.36; −0.05], *R*^2^ = 0.04. Also, the effect of self-compassion on life satisfaction was significant, *b* = 0.36, *SE* = 0.99, *t*(161) = 3.65, *p* = 0.0003, 95% CI [1.68; 5.62], *R*^2^ = 0.11. In the model that included both prospective anxiety and self-compassion as predictors of participants’ life satisfaction, prospective anxiety did not emerge as a significant predictor of life satisfaction, b = −0.08, SE = 0.08, *t*(161) = −1.02, *p* = 0.30, 95% CI [−0.25;0.07]. The direct effect of prospective anxiety on participants’ life satisfaction was not significant in this model, *b* = −0.08, *SE* = 0.08, 95% CI [−0.25;0.07], but the indirect effect was significant: *b* = −0.12, *SE* = 0.03, 95% CI [−0.20; −0.05]. Therefore, our results suggested a total mediating effect of self-compassion on the relationship between participants’ prospective anxiety and life satisfaction (see Figure 1).

*b. The mediating role of self-compassion on the relationship between participants’ inhibitory anxiety and life satisfaction.*

**FIGURE 2:**
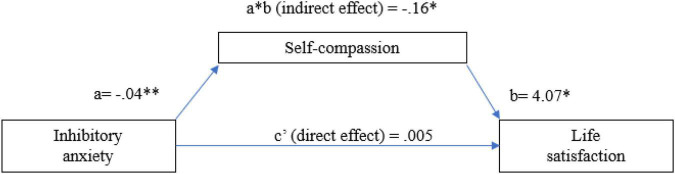
The mediating effect of self-compassion on the relationship between inhibitory anxiety and life satisfaction. The values represent unstandardized coefficients. *p < 0.05; ^**^p < 0.001; *N* = l64.

The total effect of participants’ inhibitory anxiety (i.e., without considering the mediator) was not significant; *b* = −0.16, SE = 0.10, *t(*162) = −1.48, *p* = 0.13, 95%CI [−0.37;0.05], *R*^2^ = 0.01. The effect of self-compassion on life satisfaction was significant, *b* = 4.07, *SE* = 0.98, *t*(161) = 4.13, *p* = 0.0001, 95% CI [2.13; 6.02], *R*^2^ = 0.11. In the model that included both the inhibitory anxiety and self-compassion and as predictors of participants’ life satisfaction, inhibitory anxiety did not emerge as a significant predictor of life satisfaction, b = −0.08, SE = 0.005, *t*(161) = 0.05, *p* = 0.95, 95% CI [−0.21;0.22]. The direct effect of prospective anxiety on participants’ life satisfaction was not significant in this model, *b* = −0.005, *SE* = 0.11, 95% CI [−0.21;0.22], but the indirect effect was significant: *b* = −0.16, *SE* = 0.05, 95% CI [−0.27; −0.07]. Therefore, our results suggested a total mediating effect of self-compassion on the relationship between participants’ inhibitory anxiety and life satisfaction (see Figure 2).

## Discussion

Our study investigated the indirect effect of self-compassion on the relationship between two dimensions of intolerance of uncertainty (i.e., prospective and inhibitory anxiety) and life satisfaction in the fall of 2021 when the Delta wave of COVID-19 was at its peak. People’s life satisfaction has been shown to be decreased by IU in previous studies, such as the one conducted by Deniz (2021). However, the dynamics of the link between IU, self-compassion, and life satisfaction are not yet clear at the peak of the deadliest pandemic wave in Romania, and thus, we explored the potentially significant link between prospective and inhibitory anxiety and life satisfaction, through self-compassion.

Our findings suggested significant negative associations between prospective anxiety self-compassion and life satisfaction, as well as between inhibitory anxiety and self-compassion. Thus, our findings align with previous research that highlighted these associations (e.g., Barnard and Curry, 2011; Asli Azad et al., 2019; Tang, 2019; Satici et al., 2020; Deniz, 2021). Furthermore, following mediation analyses, our results suggested a total mediating effect of self-compassion on the relationship between participants’ prospective and inhibitory anxiety and their life satisfaction. Interestingly, inhibitory anxiety was not significantly associated with life satisfaction but was significantly and negatively correlated with self-compassion.

Our findings suggested that the direct effects of both prospective and inhibitory anxiety were not significant, but their indirect effects, through self-compassion, were significant. These findings underline the importance of self-compassion as a valuable internal resource to increase one life’s satisfaction. Self-compassion promotes acceptance and positive thinking even during challenging times and personal failures, enhancing resilience, and promoting healthy coping mechanisms. Thus, intervention strategies during challenging times (and other positive or negative contexts as well) should focus more on the various ways through which self-compassion can be cultivated and promoted. For example, the Compassionate Mind Training (CMT) intervention is based on self-compassion, and it was developed to decrease depression, feelings of inferiority, or shame (Gilbert, 2009). Other interventions and therapeutical approaches based on the self-compassion facets, such as mindfulness, might also be useful models (e.g., The Mindfulness-Based Stress Reduction program developed by Kabat-Zinn, 1991; Shapiro et al. (2007); the Mindful Self-Compassion program developed by Neff, 2011).

One particularly interesting result was related to the fact that inhibitory anxiety, unlike prospective anxiety, was not significantly correlated with life satisfaction. In other words, the tendency to focus on future events to prepare for potential threats (prospective anxiety) was significantly and negatively related to life satisfaction (i.e., the higher the anxiety, the lower the life satisfaction). Meanwhile, the tendency to inhibit one’s response in an uncertain situation and the feeling of being incapable of productively responding when dealing with the unknown (Fourtounas and Thomas, 2016), were not significantly linked to life satisfaction. One of the potential explanations might be related to the COVID-19 context and the Delta wave that was at its peak when participants answered our questions. For example, participants’ focus on the potential threat of COVID-19 might have decreased their life satisfaction given the high number of deaths related to the coronavirus, the social, economic, goal, and personal disruptions, and the general psychological disequilibrium during the COVID-19 health crisis (Zhang and Fan, 2022). At the same time, inhibiting their responses to the COVID-19 crisis (inhibitory anxiety) had a smaller effect, and one of the potential explanations might be related to the dynamic link between action and emotional survival during the COVID-19 crisis. Nevertheless, there are several other potential explanations that future studies might want to explore, related, for example, to the sensorimotor pathways as control systems of transdiagnostic anxiety through IU (Goldstein Ferber et al., 2021).

Several limitations need to be addressed for the current research. First, our convenient sample size was relatively small, and future studies might benefit from exploring the links between the primary variables in more extensive and more heterogeneous samples of participants. Furthermore, convenient sampling is also a limitation because it lowers the generalizability of our findings (Crossman, 2018). Second, all the measures we used were self-reported, increasing the possibility of desirable answers. Future studies might benefit from using alternative measurements, such as experimental approaches, from decreasing this risk. Third, as we previously mentioned, the context of our research implied a highly stressful time, i.e., the peak of the Delta COVID-19 wave, when Romania faced the highest COVID-related mortality rate in the world. Therefore, our results should be interpreted with caution, given this specific ecological perspective.

However, despite these limitations, we believe that our findings might contribute to a better understanding of the various mechanisms underlying our reactions during stressful times and challenging situations. Though further studies are needed to explore these results further—using larger samples and different contextual approaches (e.g., after the pandemic is over), the current results might be integrated into specific interventions that would use self-compassion as an effective strategy to increase life satisfaction and address the psychological distress caused by intolerance of uncertainty.

## Data Availability Statement

The raw data supporting the conclusions of this article will be made available by the authors, without undue reservation.

## Ethics Statement

The studies involving human participants were reviewed and approved by the Faculty of Psychology and Educational Sciences, Alexandru Ioan Cuza University. The patients/participants provided their written informed consent to participate in this study.

## Author Contributions

Both authors contributed equally to conceiving and designing the study’s primary goal, analyzing the data, and writing the manuscript.

## Conflict of Interest

The authors declare that the research was conducted in the absence of any commercial or financial relationships that could be construed as a potential conflict of interest.

## Publisher’s Note

All claims expressed in this article are solely those of the authors and do not necessarily represent those of their affiliated organizations, or those of the publisher, the editors and the reviewers. Any product that may be evaluated in this article, or claim that may be made by its manufacturer, is not guaranteed or endorsed by the publisher.

## References

[B1] Allen A. B. Leary M. R. (2010). Self-Compassion, stress, and coping. *Soc. Pers. Psychol. Compass* 4 107–118. 10.1111/j.1751-9004.2009.00246.x 20686629 PMC2914331

[B2] Asli Azad M. Manshaei G. R. Ghamarani A. (2019). The effect of mindfulness therapy on tolerance of uncertainty and thought-action fusion in patients with obsessive-compulsive disorder. *Q. J. Child Ment. Health* 6 83–94. 10.29252/JCMH.6.1.8

[B3] Barnard L. K. Curry J. F. (2011). Self-compassion: conceptualizations, correlates, & interventions. *Rev. Gen. Psychol.* 15 289–303. 10.1037/a0025754

[B4] Benke C. Autenrieth L. K. Asselmann E. Pané-Farré C. A. (2022). One year after the COVID-19 outbreak in Germany: long-term changes in depression, anxiety, loneliness, distress and life satisfaction. *Eur. Arch. Psychiatry Clin. Neurosci.* 1–11. 10.1007/s00406-022-01400-0 35348855 PMC8962282

[B5] Birrell J. Meares K. Wilkinson A. Freeston M. (2011). Toward a definition of intolerance of uncertainty: a review of factor analytical studies of the Intolerance of Uncertainty Scale. *Clin. Psychol. Rev.* 31 1198–1208. 10.1016/j.cpr.2011.07.009 21871853

[B6] Boswell J. F. Thompson-Hollands J. Farchione T. J. Barlow D. H. (2013). Intolerance of uncertainty: a common factor in the treatment of emotional disorders. *J. Clin. Psychol.* 69 630–645. 10.1002/jclp.21965 23381685 PMC3712497

[B7] Bottesi G. Ghisi M. Carraro E. Barclay N. Payne R. Freeston M. H. (2016). Revising the intolerance of uncertainty model of generalized anxiety disorder: evidence from UK and Italian undergraduate samples. *Front. Psychol.* 7:1723. 10.3389/fpsyg.2016.01723 27847496 PMC5088195

[B8] Carleton R. N. (2012). The intolerance of uncertainty construct in the context of anxiety disorders: theoretical and practical perspectives. *Expert Rev. Neurother.* 12 937–947. 10.1586/ern.12.82 23002938

[B9] Carleton R. N. Norton M. A. P. J. Asmundson G. J. G. (2007). Fearing the unknown: a short version of the intolerance of uncertainty scale. *J. Anxiety Disord.* 21 105–117. 10.1016/j.janxdis.2006.03.014 16647833

[B10] Chen S. Yao N. Qian M. (2018). The influence of uncertainty and intolerance of uncertainty on anxiety. *J. Behav. Ther. Exp. Psychiatry* 61 60–65. 10.1016/j.jbtep.2018.06.005 29909250

[B11] Crossman A. (2018). *Convenience Samples for Research.* Thought Co. Available online at: http://www.thoughtco.com/convenience-sampling-3026726 (accessed April 2022).

[B12] Del-Valle M. V. López-Morales H. Andrés M. L. Yerro-Avincetto M. Gelpi Trudo R. Urquijo S. (2022). Intolerance of COVID-19-related uncertainty and depressive and anxiety symptoms during the pandemic: a longitudinal study in Argentina. *J. Anxiety Disord.* 86:102531. 10.1016/j.janxdis.2022.102531 35066351 PMC8750696

[B13] Deniz M. E. (2021). Self-compassion, intolerance of uncertainty, fear of COVID-19, and well-being: a serial mediation investigation. *Pers. Individ. Dif.* 177:110824. 10.1016/j.paid.2021.110824 33723469 PMC7945866

[B14] Diener E. Emmons R. A. Larsen R. J. Griffin S. (1985). The satisfaction with life scale. *J. Pers. Assess.* 49 71–75. 16367493 10.1207/s15327752jpa4901_13

[B15] Diener E. Suh E. M. Lucas R. E. Smith H. L. (1999). Subjective well-being: three decades of progress. *Psychol. Bull.* 125 276–302.

[B16] Dugas M. J. Gagnon F. Ladouceur R. Freeston M. H. (1998). Generalized anxiety disorder: a preliminary test of a conceptual model. *Behav. Res. Ther.* 36 215–226. 10.1016/s0005-7967(97)00070-3 9613027

[B17] Fourtounas A. Thomas S. J. (2016). Cognitive factors predicting checking, procrastination and other maladaptive behaviours: prospective versus Inhibitory Intolerance of Uncertainty. *J. Obsessive Compuls. Relat. Disord.* 9 30–35.

[B18] Gherasim C. (2021). *Romania Reaches Historic High in Covid Deaths.* Available online at: https://euobserver.com/coronavirus/153428 (accessed April 2022).

[B19] Gilbert P. (2009). *The Compassionate Mind.* London: Constable.

[B20] Goetz J. L. Keltner D. Simon-Thomas E. (2010). Compassion: an evolutionary analysis and empirical review. *Psychol. Bull.* 136 351–374. 10.1037/a0018807 20438142 PMC2864937

[B21] Goldstein Ferber S. Shoval G. Zalsman G. Mikulincer M. Weller A. (2021). Between action and emotional survival during the COVID-19 era: sensorimotor pathways as control systems of transdiagnostic anxiety-related intolerance to uncertainty. *Front. Psychiatry* 12:680403. 10.3389/fpsyt.2021.680403 34393847 PMC8358206

[B22] Hayes A. F. (2013). *Introduction to Mediation, Moderation, and Conditional Process Analysis: A Regression-Based Approach.* New York, NY: Guilford Press.

[B23] Jayaram S. Krishnamurthy P. T. Selvaraj D. Nemani S. V. Rymbai E. Sugumar D. (2022). Temporal unpredictability and probabilistic uncertainty induced anxiety in the times of COVID-19 pandemic. *Indian J. Pharm. Educ. Res.* 56 321–328.

[B24] Kabat-Zinn J. (1991). *Full Catastrophe Living: Using the Wisdom of Your Body and Mind to Face Stress, Pain, and Illness.* New York, NY: Dell Publishing.

[B25] Karataş Z. Tagay Ö. (2021). The relationships between resilience of the adults affected by the covid pandemic in Turkey and Covid-19 fear, meaning in life, life satisfaction, intolerance of uncertainty and hope. *Pers. Individ. Dif.* 172:110592. 10.1016/j.paid.2020.110592 33518871 PMC7832104

[B26] Kim C. Ko H. (2018). The impact of self-compassion on mental health, sleep, quality of life and life satisfaction among older adults. *Geriatr. Nurs.* 39 623–628. 10.1016/j.gerinurse.2018.06.005 30054094

[B27] Kim H. Y. (2013). Statistical notes for clinical researchers: assessing normal distribution (2) using skewness and kurtosis. *Restor. Dent. Endod.* 38 52–54. 10.5395/rde.2013.38.1.52 23495371 PMC3591587

[B28] Kozina A. Peras I. Veldin M. Pivec T. (2022). The psychological response and perception of stress during the COVID-19 pandemic in slovenia: Three-wave repeated cross-sectional study. *Stress Health* 10.1002/smi.3147 35338675 PMC9111042

[B29] Kunzler A. M. Röthke N. Günthner L. Stoffers-Winterling J. Tüscher O. Coenen M. (2021). Mental burden and its risk and protective factors during the early phase of the SARS-CoV-2 pandemic: systematic review and meta-analyses. *Global. Health* 17:34. 10.1186/s12992-021-00670-y 33781283 PMC8006628

[B30] Kyriazos T. A. (2018). Applied psychometrics: the 3-faced construct validation method, a routine for evaluating a factor structure. *Psychology* 9 2044–2072. 10.4236/psych.2018.98117

[B31] Lau B. H. Chan C. L. Ng S. M. (2020). Self-compassion buffers the adverse mental health impacts of COVID-19-related threats: results from a cross-sectional survey at the first peak of Hong Kong’s outbreak. *Front. Psychiatry* 11:585270. 10.3389/fpsyt.2020.585270 33250793 PMC7674650

[B32] Loewe N. Bagherzadeh M. Araya-Castillo L. Thieme C. Batista-Foguet J. M. (2014). Life domain satisfactions as predictors of overall life satisfaction among workers: evidence from Chile. *Soc. Indic. Res.* 118 71–86. 10.1007/s11205-013-0408-6 25018580 PMC4082135

[B33] Luhmann C. C. Ishida K. Hajcak G. (2011). Intolerance of uncertainty and decisions about delayed, probabilistic rewards. *Behav. Ther.* 42 378–386. 10.1016/j.beth.2010.09.002 21658521

[B34] MacBeth A. Gumley A. (2012). Exploring compassion: a meta-analysis of the association between self-compassion and psychopathology. *Clin. Psychol. Rev.* 32 545–552. 10.1016/j.cpr.2012.06.003 22796446

[B35] Maftei A. Holman A.-C. (2022). Beliefs in conspiracy theories, intolerance of uncertainty, and moral disengagement during the coronavirus crisis. *Ethics Behav.* 32 1–11. 10.1080/10508422.2020.1843171

[B36] McEvoy P. M. Mahoney A. E. Moulds M. L. (2010). Are worry, rumination, and post-event processing one and the same?: Development of the Repetitive Thinking Questionnaire. *J. Anxiety Disord*. 24, 509–519. 10.1016/j.janxdis.2010.03.008 20409676

[B37] McEvoy P. M. Mahoney A. E. (2011). Achieving certainty about the structure of intolerance of uncertainty in a treatment-seeking sample with anxiety and depression. *J. Anxiety Disord.* 25 112–122. 10.1016/j.janxdis.2010.08.010 20828984

[B38] McEvoy P. M. Mahoney A. E. (2012). To be sure, to be sure: intolerance of uncertainty mediates symptoms of various anxiety disorders and depression. *Behav. Ther.* 43 533–545. 10.1016/j.beth.2011.02.007 22697442

[B39] Millroth P. Frey R. (2021). Fear and anxiety in the face of COVID-19: negative dispositions towards risk and uncertainty as vulnerability factors. *J. Anxiety Disord.* 83:102454. 10.1016/j.janxdis.2021.102454 34298237 PMC8426312

[B40] Neff K. D. (2011). Self-compassion, self-esteem, and well-being. *Soc. Pers. Psychol. Compass* 5 1–12. 10.1111/j.1751-9004.2010.00330.x

[B41] Neff K. D. Germer C. (2017). “Chap. 27 - Self-compassion and psychological wellbeing,” in *Oxford Handbook of Compassion Science*, ed. Doty J. (Oxford: Oxford University Press).

[B42] Neff K. Knox M. C. (2016). “Self-compassion: embracing suffering with kindness,” in *Mindfulness in Positive Psychology: The Science of Meditation and Well-Being*, eds Ivtzan I. Lomas T. (Abingdon: Routledge), 37–50.

[B43] Osmanaðaoðlu N. Creswell C. Dodd H. F. (2018). Intolerance of Uncertainty, anxiety, and worry in children and adolescents: a meta-analysis. *J. Affect. Disord.* 225 80–90. 10.1016/j.jad.2017.07.035 28802117

[B44] Ouellet C. Langlois F. Provencher M. D. Gosselin P. (2019). Intolerance of uncertainty and difficulties in emotion regulation: proposal for an integrative model of generalized anxiety disorder. *Eur. Rev. Appl. Psychol.* 69 9–18. 10.1016/j.erap.2019.01.001

[B45] Pavot W. Diener E. (2008). The Satisfaction With Life Scale and the emerging construct of life satisfaction. *J. Posit. Psychol.* 3 137–152. 10.1080/17439760701756946

[B46] Racine S. Miller A. Mehak A. Trolio V. (2022). Examining risk and protective factors for psychological health during the COVID-19 pandemic. *Anxiety Stress Coping* 35 124–140. 10.1080/10615806.2021.1958789 34314272

[B47] Raes F. Pommier E. Neff K. D. Van Gucht D. (2011). Construction and factorial validation of a short form of the Self-Compassion Scale. *Clin. Psychol. Psychother.* 18 250–255. 10.1002/cpp.702 21584907

[B48] Rettie H. Daniels J. (2021). Coping and tolerance of uncertainty: predictors and mediators of mental health during the COVID-19 pandemic. *Am. Psychol.* 76 427–437. 10.1037/amp0000710 32744841

[B49] Satici B. Saricali M. Satici S. A. Griffiths M. D. (2020). Intolerance of uncertainty and mental well-being: serial mediation by rumination and fear of COVID-19. *Int. J. Ment. Health Addict.* 1–12. 10.1007/s11469-020-00305-0 32427165 PMC7228430

[B50] Shapiro S. L. Brown K. W. Biegel G. M. (2007). Teaching self-care to caregivers: effects of mindfulness-based stress reduction on the mental health of therapists in training. *Train. Educ. Prof. Psychol*. 1, 105–115. 10.1037/1931-3918.1.2.105

[B51] Souza L. K. D. Hutz C. S. (2016). Self-compassion in relation to self-esteem, self-efficacy and demographical aspects. *Paidéia (Ribeirão Preto)* 26 0181–0188. 10.1590/1982-43272664201604

[B52] Soysa C. K. Wilcomb C. J. (2015). Mindfulness, self-compassion, self-efficacy, and gender as predictors of depression, anxiety, stress, and well-being. *Mindfulness* 6 217–226. 10.1007/s12671-013-0247-1

[B53] Tang W. K. (2019). Resilience and self-compassion related with achievement emotions, test anxiety, intolerance of uncertainty, and academic achievement. *Psychol. Stud*. 64, 92–102. 10.1007/s12646-019-00482-6

[B54] Thibodeau M. A. Carleton R. N. Gómez-Pérez L. Asmundson G. J. (2013). ”What if I make a mistake?”: intolerance of uncertainty is associated with poor behavioral performance. *J. Nerv. Ment. Dis.* 201 760–766. 10.1097/nmd.0b013e3182a21298 23995031

[B55] Tyupa S. (2011). A theoretical framework for back-translation as a quality assessment tool. *New Voices Transl. Stud.* 7 35–46. 10.1080/09638288.2021.2003452 34802337

[B56] Voitsidis P. Nikopoulou V. A. Holeva V. Parlapani E. Sereslis K. Tsipropoulou V. (2021). The mediating role of fear of COVID-19 in the relationship between intolerance of uncertainty and depression. *Psychol. Psychother.* 94 884–893. 10.1111/papt.12315 33216444 PMC7753422

[B57] Worldmeter (2022). *COVID-19 Coronavirus Pandemic.* Available online at: https://www.worldometers.info/coronavirus/ (accessed April 2022).

[B58] Wu X. Nazari N. Griffiths M. D. (2021). Using fear and anxiety related to COVID-19 to predict cyberchondria: cross-sectional survey study. *J. Med. Internet Res.* 23:e26285. 10.2196/26285 34014833 PMC8191728

[B59] Yang Y. Zhang M. Kou Y. (2016). Self-compassion and life satisfaction: the mediating role of hope. *Pers. Individ. Dif.* 98 91–95. 10.1016/j.paid.2016.03.086

[B60] Zhang Q. Fan J. (2022). Goal disruption and psychological disequilibrium during the outbreak of COVID-19: the roles of uncertainty, information seeking and social support. *Health Commun*. 1–8. 10.1080/10410236.2022.2049046 35282726

